# Comparing Selective Serotonin Reuptake Inhibitors (SSRIs) Alone and in Combination With Beta-Blockers for Treating Panic Disorders: A Prospective Cohort Study

**DOI:** 10.7759/cureus.68862

**Published:** 2024-09-07

**Authors:** Doaa Bajabir, Abdullah Alsubhi, Saja A Felimban, Ruba Z Alotaibi, Aisha Almalki, Nawaf S Allahyani, Raghad Y Yaseen, Feras B Kofiah, Abdulaziz A Almatrafi, Saif A Alzahrani

**Affiliations:** 1 Psychiatry, Medical Cities Program, Ministry of Interior, Riyadh, SAU; 2 Medicine, Umm Al-Qura University, Makkah, SAU; 3 Medicine and Surgery, Umm Al-Qura University, Makkah, SAU; 4 Psychiatry, Umm Al-Qura University, Makkah, SAU; 5 Medicine and Surgery, Umm Al-Qura University, Al-Qunfudah, SAU; 6 Preventive Medicine, Ministry of National Guard Health Affairs, Jeddah, SAU

**Keywords:** anxiety, combination therapy, comorbidities, depression, pharmacotherapy, symptom severity

## Abstract

Background

Panic disorders are prevalent psychiatric conditions affecting 1.6% to 2.2% of the global population. While selective serotonin reuptake inhibitors (SSRIs) are the first line of treatment, their initial exacerbation of symptoms presents challenges. Beta-blockers have shown promise in managing panic symptoms, but research comparing the efficacy of combined SSRI and beta-blocker therapy to SSRI monotherapy is limited, particularly in Saudi Arabia.

Objective

To assess the effectiveness of SSRIs combined with beta-blockers vs. SSRI monotherapy in improving panic disorder symptoms severity in patients at King Abdul-Aziz Hospital, Makkah, Saudi Arabia.

Methods

This prospective cohort study included 62 patients with panic disorder, divided into two groups: SSRI monotherapy (n=29) and SSRIs with beta-blockers (n=33). Panic disorder severity was assessed using the Panic Disorder Severity Scale (PDSS) after three months of treatment. Secondary outcomes included depression and anxiety symptoms, measured by the Patient Health Questionnaire (PHQ-9) and General Anxiety Disorder Scale (GAD-7), respectively. Statistical analysis involved Mann-Whitney U tests for comparing PDSS scores between the groups due to non-parametric distribution and Chi-square tests for categorical variables. Relative risks (RR) were calculated to assess the likelihood of abnormal PDSS, PHQ-9, and GAD-7 scores between the groups. Multivariable linear regression was used to adjust for potential confounding factors.

Results

No statistically significant difference in PDSS scores was found between SSRI monotherapy (median=6, interquartile range (IQR)=3-9) and combination therapy (median=8, IQR=3-13) groups (p=0.188). The relative risk of abnormal PDSS scores was 1.8 times higher in the combination therapy group (p=0.077). No significant differences in depression (p=0.386) or anxiety (p=0.182) symptoms were observed. Additionally, 66.7% of combination therapy patients had abnormal PDSS scores compared to 33.3% in the SSRI group. The mean PHQ-9 score was 11.08±6.93, and the mean GAD-7 score was 10.69±6.41 for the total sample.

Conclusion

This study found no significant difference in the effectiveness of SSRIs combined with beta-blockers vs. SSRI monotherapy for treating panic disorders. However, the trend towards higher PDSS scores in the combination therapy group suggests further investigation is needed. Study limitations included small sample size, single-center design, short follow-up period, and lack of randomization. Despite these, the study provided valuable insights into treatment approaches for panic disorders in the Saudi population. Larger, randomized controlled trials with longer follow-up periods and multi-center designs are recommended for future research.

## Introduction

Panic disorders are prevalent psychiatric conditions affecting approximately 1.6-2.2% of the global population, with serious implications for mental health [1], and a one-year and lifetime occurrence of 2.4% and 3.8%, respectively [2]. The prevalence of panic disorder is highest during adolescence and early adulthood, while it is relatively low in children aged below 14 years [3]. In Saudi Arabia, panic disorder exhibits a lifetime prevalence of 1.6%, with an 82.6% persistence rate over 12 months, underscoring the chronic nature of the disorder [4].

According to the Diagnostic and Statistical Manual of Mental Disorders (DSM), panic disorder is characterized by sudden and acute episodes of fear or discomfort known as panic attacks. Symptoms involve rapid heartbeat, shortness of breath, chest discomfort, sweating, and a sense of disorientation that usually peaks within minutes [5]. Panic disorder is characterized by recurring, spontaneous panic episodes compared to those associated with social anxiety disorder, which are triggered by specific social events [6]. The lifetime treatment rate of panic disorder in the Saudi National Mental Health Survey (SNMHS) is 49.7%, and the rate of treatment in the year of onset is 7.8%, which is the highest rate preceded by generalized anxiety disorder, bipolar disorder, and major depressive disorder [7]. The etiology of panic disorder is multifactorial, involving both environmental and genetic influences, contributing to its complexity and treatment challenges [8].

According to the American Psychiatric Association guidelines, psychosocial and pharmacological interventions are the primary options for the treatment of panic disorder, with selective serotonin receptor inhibitors (SSRIs) commonly suggested as first-line medication [2]. SSRIs, commonly prescribed for panic disorder, are effective but may initially exacerbate symptoms before producing therapeutic benefits, whereas benzodiazepines respond immediately but have tolerance issues; however, comparisons between the two are rare [1]. Propranolol, a pioneering beta-blocker, penetrates the blood-brain barrier and impacts both somatic and central nervous system tissues [9]. It is commonly administered orally, with doses ranging from 10-80 mg per dose in the form of Inderal [10]. Daily oral propranolol has demonstrated efficacy in suppressing panic attacks in individuals diagnosed with panic disorder. In comparison to other beta-blockers like oxprenolol, propranolol has shown greater effectiveness in reducing palpitations and lowering heart rate [11].

While few studies have examined the general interaction between beta-blockers and SSRIs, most research has focused on specific medications rather than the broader drug classes. For instance, studies have investigated the interactions between particular paroxetine and metoprolol, providing more detailed insights into their combined effects. Paroxetine was found to inhibit the metabolism of metoprolol, resulting in increased metoprolol plasma concentrations. This interaction may lead to exaggerated therapeutic effects and adverse events, particularly in patients with cardiovascular conditions [12]. The combination of paroxetine and metoprolol has been associated with a risk of hemodynamic adverse events in patients with cardiovascular disease. To mitigate these risks, it is crucial to carefully monitor heart rate and blood pressure, especially when initiating or adjusting beta-blocker therapy in patients concurrently receiving paroxetine [13].

Despite the prevalence of panic disorders and their significant impact on quality of life, there is limited research in Saudi Arabia comparing the efficacy of SSRIs combined with beta-blockers vs. SSRI monotherapy in treating these conditions. This study aims to address this gap by investigating the effectiveness of SSRIs in combination with beta-blockers compared to SSRI monotherapy in improving panic disorder symptoms severity. We hypothesize that individuals with panic disorder who receive a combination of SSRIs and beta-blockers will experience a greater reduction in panic disorder symptom severity compared to those who receive SSRI monotherapy. This hypothesis is based on the potential complementary mechanisms of action of these two drug classes in addressing different aspects of panic disorder symptomatology.

## Materials and methods

Study design and setting

This study employed a prospective cohort design conducted at King Abdul-Aziz Hospital in Makkah, Saudi Arabia, ensuring consistency in the study environment. All subjects were drawn from this facility to ensure consistency in the study environment and minimize confounding variables. The study continued for one year beginning in January 2022 after receiving ethical permission. The primary selection of the participants was based on the therapeutic regimen (SSRIs and beta-blockers vs. SSRI monotherapy). An outcome assessment was done for patients after completing three months of their treatment.

Outcome assessment

The Panic Disorder Severity Scale (PDSS) was used to assess the outcome of the current study [14]. The PDSS is a validated instrument designed to measure the severity of panic disorder symptoms. It consists of seven items, each rated on a five-point Likert Scale ranging from 0 (none) to 4 (extreme). The PDSS evaluates various aspects of panic disorder, including frequency of panic attacks, distress during attacks, anticipatory anxiety, agoraphobic fear and avoidance, fear and avoidance of physical sensations, and work and social impairment. Higher scores on the PDSS indicate greater severity of panic disorder symptoms. 

Participant selection

Calculations were made using an online tool (openepi.com) for cohort studies. The equation was built with a 95% confidence level (95% CI), 80% power, and estimated relative risk of four. With an unexposed to exposed ratio of 1:1, the total calculated sample size was 64 participants.

In our study, people with panic disorders who were following up at the outpatient clinics of King Abdul-Aziz Hospital in Makkah were considered for inclusion. The study population included all panic disorder patients who visited the outpatient clinic at King Abdul-Aziz Hospital in Makkah. Participants were categorized into two groups: those receiving combination therapy with SSRIs and beta-blockers and those on SSRI monotherapy. Inclusion criteria included ages above 18 years of both genders and following up at King Abdul-Aziz Hospital clinics. Patients with mental retardation, cognitive impairment, epilepsy, and those taking hypnotic or sedative drugs such as benzodiazepines were excluded from the study.

Data collection

The daily clinical patients log and medical records system were used to obtain the information of eligible patients. The information of patients matching the inclusion criteria was drawn, and they were invited to participate in the study. Data were collected via structured phone interviews, ensuring a comprehensive capture of demographic and clinical variables. Collected data included sociodemographic variables (e.g., age, gender, weight, height, educational level, and income) and validated tools to assess the outcome and potential confounding mental illnesses. The tools included PDSS to assess the panic disorder severity [14], the Patient Health Questionnaire (PHQ-9) to assess depression [15], and the Generalized Anxiety Disorder Scale (GAD-7) to assess anxiety [15].

The Arabic version of PHQ-9 and GAD-7 were validated previously after translation from the original English version [15]. The Arabic version of the PDSS tool was not available, so we followed the scientific method for translation. This process involved forward translation to Arabic by an Arabic expert fluent in English, followed by backward translation to English by a native English speaker fluent in Arabic [16]. Discrepancies between the final English version and the original PDSS version were addressed through modifications to the Arabic version. While we did not conduct a formal validation study due to it being outside the scope of our research, we ensured content validity through a review by experts in the field of psychiatry. The reliability of the questionnaire was tested among the study sample using Cronbach's alpha and showed a reliability score of (r=0.90). 

Statistical analysis

Data were analyzed using IBM SPSS Statistics (IBM Corp. Released 2023. IBM SPSS Statistics for Mac, Version 29.0.2.0, Armonk, NY: IBM Corp), with appropriate tests selected based on data distribution and study design. No missing values were identified in the analyzed data set. Descriptive statistics were used to summarize the demographic data and clinical characteristics of the study participants. Continuous variables were presented as means and standard deviations (SD) or medians and interquartile ranges (IQR), depending on the distribution of the data. Categorical variables were presented as frequencies and percentages.

Due to skewed distribution, the Mann-Whitney U test was used to compare continuous variables between the two treatment groups (SSRIs and beta-blockers vs. SSRI monotherapy). Chi-square tests were used to compare categorical variables between the two groups, while Fisher's exact tests were employed when Chi-square assumptions were not met. PDSS scores between the groups were compared using the Mann-Whitney U test considering the non-parametric nature of the PDSS scores.

Relative risks (RR) were calculated to investigate the risk of depressive symptoms, anxiety symptoms, and panic disorder symptoms between the two treatment groups. A p-value of less than 0.05 was considered statistically significant. To assess the impact of potential confounding factors, such as PHQ-9 and GAD-7 scores, multivariable analysis was conducted using linear regression. The results were reported as adjusted odds ratios (OR) or beta coefficients with 95% CI. Subgroup analyses were conducted to explore potential differences in treatment effects based on gender, depression status, and anxiety status. These analyses were performed using the Mann-Whitney U test and linear regression and were considered statistically significant at (0.05). However, due to the relatively small sample size, most comparisons did not reveal significant differences between the treatment groups.

Ethical considerations

Ethical approval was obtained from the Institutional Review Board (IRB) at the Directorate of Health Affairs affiliated with the Ministry of Health, Makkah, Saudi Arabia (IRB approval number: H-02-K-076-0123-886). Patient contact information and names were acquired from the hospital records system after obtaining IRB approval and institution permission. Verbal consent was obtained from all participants after explaining the study's nature and purposes. The decision to use verbal consent instead of written consent was made to facilitate the data collection process, considering the study's low-risk nature and the potential logistical challenges in obtaining written consent during phone interviews. This approach also helped to maintain participant anonymity and reduce potential barriers to participation. All acquired data was securely stored and used for this research purpose only.

## Results

Out of the total sample of 62 patients diagnosed with panic disorder on treatment, 33 (46.8%) patients were taking SSRIs alone, while 29 (53.2%) were taking SSRIs and beta-blockers. Regarding gender, 34 (54.8%) participants were males. The distribution of marital status showed that the majority of participants, that is, 44 (71%) were married, followed by 10 (16.1%) single individuals. In terms of educational level, the highest proportion of participants, that is, 22 (35.5%) had a university education, followed by 20 (32.3%) with high school education. Concerning personal income, 37 (59.7%) participants had an income of less than 5,000 SAR, while 11 (17.7%) had an income between 10,001 and 15,000 SAR. Smoking status showed 24 (38.7%) participants were smokers. Regarding body mass index (BMI) groups, more than one-third or 23 (37.1%) participants were overweight, followed by 18 (29%) normal weight. Additional percentages and p-values for each variable are shown in Table 1.

**Table 1:** Baseline comparison of demographic and clinical characteristics of the study participants *Percentages calculated by column SSRIs: Selective serotonin reuptake inhibitors; BMI: Body mass index

Variable	Groups	Total (%)*	Drugs		P-value
			SSRIs + beta-blockers (%)	SSRIs (%)	
Gender	Male	34 (54.8%)	20 (58.8%)	14 (41.2%)	0.237
	Female	28 (45.2%)	13 (46.4%)	15 (53.6%)	
Marital status	Single	10 (16.1%)	8 (80%)	2 (20%)	0.087
	Married	44 (71%)	22 (50%)	22 (50%)	
	Divorced	5 (8.1%)	3 (60%)	2 (40%)	
	Widow	3 (4.8%)	0 (0%)	3 (100%)	
Educational level	No official education	10 (16.1%)	6 (60%)	4 (40%)	0.991
	Elementary school	6 (9.7%)	3 (50%)	3 (50%)	
	Secondary school	3 (4.8%)	2 (66.7%)	1 (33.3%)	
	Highschool	20 (32.3%)	10 (50%)	10 (50%)	
	University	22 (35.5%)	11 (50%)	11 (50%)	
	Higher education	1 (1.6%)	1 (100%)	0 (0%)	
Personal income	< 5,000 SAR	37 (59.7%)	20 (54.1%)	17 (45.9%)	0.607
	5,001 - 10,000 SAR	10 (16.1%)	7 (70%)	3 (30%)	
	10,001 - 15,000 SAR	11 (17.7%)	4 (36.4%)	7 (63.6%)	
	> 15,000 SAR	4 (6.5%)	2 (50%)	2 (50%)	
Smoking	Smoker	24 (38.7%)	13 (54.2%)	11 (45.8%)	0.557
	Not smoker	38 (61.3%)	20 (52.6%)	18 (47.4%)	
BMI group	Underweight	0 (0%)	0 (0%)	0 (0%)	0.949
	Normal	18 (29%)	9 (50%)	9 (50%)	
	Overweight	23 (37.1%)	13 (56.5%)	10 (43.5%)	
	Obese	21 (33.9%)	11 (52.4%)	10 (47.6%)	

The sample's mean age was 42.08±11.39 years, with no significant age differences between the treatment groups. The mean BMI was 28.48±5.51. There were no significant differences in age (p=0.209) and BMI (p=0.333) between the treatment groups. The compliance was reported as a median of 10 (IQR: 5-10) similar for both groups (p=0.858). The mean PHQ-9 score across the sample was 11.08 ± 6.93, indicating moderate depressive symptoms, and the mean GAD-7 total score was 10.69±6.41 for the total sample. The PDSS raw score was reported as a median of 6.5 (IQR: 3-11) for the total sample, 6 (IQR: 3-9) for the SSRIs group, and 8 (IQR: 3-13) for the SSRIs and beta-blockers group (p=0.188). Detailed comparative results highlighting key differences and statistical significance between the groups are presented in Table 2.

**Table 2:** Comparison of continuous demographic and clinical characteristics between the treatment groups *P-value calculated using Mann-Whitney U test SSRIs: Selective serotonin reuptake inhibitors; PDSS: Panic Disorder Severity Scale; GAD-7: General Anxiety Disorder Scale; PHQ-9: Patient Health Questionnaire; BMI: Body mass index

	Total sample	SSRIs + beta-blockers	SSRIs	P-value
Age	42.1±11.4	40.9±11.4	43.3±11.5	0.209
BMI	28.5±5.5	28.2±5.4	28.8±5.7	0.333
Compliance	10 (5-10)	10 (6-10)	10 (5-10)	0.858*
PHQ-9 total score	11.1±6.9	12.1±6.9	9.9±6.8	0.119
GAD-7 total score	10.7±6.4	10.9±6.2	10.4±6.7	0.375
PDSS raw score	6.5 (3-11)	8 (3-13)	6 (3-9)	0.188*

For depression severity, 15 (24.2%) participants had no depression symptoms, 10 (16.1%) had mild symptoms, 17 (27.4%) had moderate symptoms, 13 (21%) had moderately severe symptoms, and seven (11.3%) had severe symptoms. In terms of anxiety severity, 15 (24.2%) had no anxiety symptoms, 12 (19.4%) had mild symptoms, 15 (24.2%) had moderate symptoms, and 20 (32.3%) had severe symptoms. In terms of panic disorder severity, 38 (61.3%) had normal scores, indicating no need for evaluation, while 24 (38.7%) required evaluation. 

**Figure 1:**
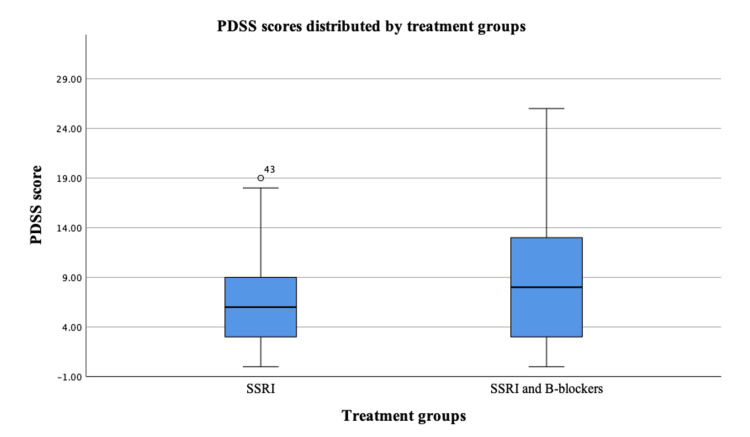
Comparison of PDSS scores between SSRIs vs. SSRIs and beta-blockers treatment groups SSRIs: Selective serotonin reuptake inhibitors; PDSS: Panic Disorder Severity Scale; B-blockers: Beta-blockers

Figure 1 shows the comparison of PDSS scores between SSRIs vs. SSRIs and beta-blockers treatment groups. The median PDSS score for the SSRIs group was 6 (IQR: 3-9), while the median PDSS score for the SSRIs and beta-blockers group was 8 (IQR: 3-13). The p-value for the comparison was 0.188. After adjusting for PHQ-9 and GAD-7 scores, the adjusted p-value remained 0.188. These results suggest that there was no statistically significant difference in PDSS scores between SSRIs vs. SSRIs and beta-blockers treatment groups.

For the PHQ-9 category, 26 (55.3%) participants in the SSRIs and beta-blockers group had abnormal scores, while 21 (44.7%) participants in the SSRIs group had abnormal scores. RR calculations indicated a minimal difference in depressive symptom prevalence between the SSRI monotherapy and combination therapy groups (RR=1.1), and the difference was not statistically significant (p=0.386). For the GAD-7 category, 27 (57.4%) participants in the SSRIs and beta-blockers group had abnormal scores, while 20 (42.6%) participants in the SSRIs group had abnormal scores. The RR between the two groups was 1.2. In terms of the PDSS category, 16 (66.7%) participants in the SSRIs and beta-blockers group had abnormal scores, while eight (33.3%) participants in the SSRIs group had abnormal scores. Despite that, the results were not statistically significant (p=0.077); RR between the two groups was 1.8. A detailed comparison of the treatment groups is shown in Table 3. 

**Table 3:** Comparison of treatment groups SSRIs vs. SSRIs and beta-blockers in terms of PHQ-9, GAD-7, and PDSS categories SSRIs: Selective serotonin reuptake inhibitors; RR: Relative risks; PDSS: Panic Disorder Severity Scale; GAD-7: General Anxiety Disorder Scale; PHQ-9: Patient Health Questionnaire

		SSRIs + beta-blockers		SSRIs		RR
						P-value
		Count	N%	Count	N%	
PHQ-9	Abnormal	26	55.30%	21	44.70%	1.1
	Normal	7	46.70%	8	53.30%	0.386
GAD-7	Abnormal	27	57.40%	20	42.60%	1.2
	Normal	6	40%	9	60%	0.189
PDSS	Abnormal	16	66.70%	8	33.30%	1.8
	Normal	17	44.70%	21	55.30%	0.077

## Discussion

This study compared the efficacy of SSRI monotherapy vs. SSRIs combined with beta-blockers in managing panic disorder symptoms and associated conditions. The results revealed no statistically significant difference in symptom severity between the two treatment approaches across measures of panic disorder severity (PDSS), depression (PHQ-9), and anxiety (GAD-7). The study involved 62 participants with a balanced gender distribution, of which 61.30% showed normal panic disorder severity scores, while 38.70% required further evaluation. These findings challenge the hypothesis that adding beta-blockers to SSRI treatment would provide superior outcomes and suggest that clinicians may not need to routinely combine these medications for panic disorder patients. However, the presence of a subset of patients requiring further evaluation emphasizes the need for personalized treatment strategies and additional research to identify specific patient subgroups that might benefit more from one treatment modality over the other.

Previous literature supports the efficacy of antidepressants, including SSRIs, over placebo in adult patients, though the benefits of adjunctive therapies like beta-blockers remain inconclusive [17]. On the other hand, investigations of beta-blockers have suggested good efficacy in treating performance anxiety and panic disorders [18]. Steenen et al. suggested in a meta-analysis that propranolol and benzodiazepines had no statistically significant differences in efficacy in the short-term treatment of panic disorder. However, other studies in the meta-analysis tend to support the use of beta-blockers as a supplement rather than a single therapy [19]. Shea and Feero's recommendations align with the findings supporting the use of beta-blockers like pindolol in conjunction with SSRIs for treatment-resistant panic disorder, though our study did not find significant benefits in this combination [20]. 

Additionally, a small randomized controlled trial demonstrated significant clinical improvements when pindolol was added to fluoxetine, as opposed to fluoxetine alone, across various measures of outcome variables, including the Hamilton Rating Scale for Anxiety, Panic Self Questionnaire, and Clinical Anxiety Scale Plus Panic Attacks [21]. In a systematic review by Holt and Lydiard, multiple treatment options for panic disorder were examined. These included the use of SSRIs, enhancing SSRIs with beta-blockers, and combining two different classes of antidepressants. Similar to our results, there was no statistically significant difference between using SSRIs alone or augmented with beta-blockers. However, studies on combinations of different classes of antidepressants have shown promising outcomes, which would require further investigations [22]. 

With a focus on the relationship between beta-blocker use and mental health outcomes, Yasmina Molero's Swedish study employed a population-based longitudinal cohort methodology. The study's large sample size and thorough statistical analysis did not support the correlation between beta-blockers and changes in the treatment outcome of psychiatric diseases. However, the same study reported a reduction in violent behavior linked to beta-blocker use, implying the effectiveness of beta-blockers [23]. In contrast, our study, which specifically targeted panic disorder, found no significant benefits of adding beta-blockers to SSRI treatment. Thus, while beta-blockers may influence behaviors associated with other psychiatric disorders, our results suggest they don't have the same impact on panic disorder and panic attacks. Further research would be needed to fully understand these differences [23].

Our results have several important implications for the treatment of panic disorders. Firstly, the comparable efficacy observed between SSRI monotherapy and SSRIs combined with beta-blockers suggests that clinicians may not need to routinely prescribe beta-blockers as an adjunct therapy for panic disorder patients. This could potentially simplify treatment regimens, reduce medication costs, and minimize the risk of side effects associated with polypharmacy. However, the presence of a subset of patients requiring further evaluation (38.70%) indicates that neither treatment approach is universally effective, emphasizing the need for personalized treatment strategies. This finding underscores the importance of individualized patient assessment and tailored treatment plans in managing panic disorders. Additionally, the study's results highlight the need for further research to identify specific patient subgroups that might benefit more from one treatment modality over the other, potentially leading to more targeted and effective treatment approaches in the future. Lastly, these findings contribute to the ongoing discussion about the optimal use of pharmacological interventions in panic disorder management, potentially influencing clinical guidelines and decision-making processes in psychiatric care.

This study contributes to our understanding of panic disorder treatment outcomes, but its limitations, including the small sample size and lack of long-term follow-up, should be acknowledged. Our study was conducted in a single hospital. While this approach minimizes confounding variables, it might obscure the true effects of beta-blockers. Nonetheless, the current study accounts for co-illnesses such as depression and anxiety as confounders in the statistical analysis. This lends further validation to our results. An additional strength of this study is the use of well-validated outcome measures, which enhance the reliability and generalizability of our findings.

## Conclusions

In conclusion, our study provides insights into the treatment outcomes of patients with panic disorder, comparing SSRI monotherapy and combination therapy with beta-blockers. While variations in symptom prevalence were observed between the two treatment groups, none of these differences reached statistical significance, indicating comparable efficacy. Our empirical observations suggest no statistically significant discrepancies between SSRI monotherapy and combination therapy with beta-blockers in relation to the prevalence of depressive, anxiety, and panic disorder symptoms, emphasizing the need for individualized treatment approaches. Given these findings, it is crucial for clinicians to consider individual patient profiles and preferences when selecting treatment options for panic disorder. The comparable outcomes observed in our study underscore the importance of tailoring treatment to each patient's specific needs and responses. This personalized approach may lead to better patient compliance and overall treatment success. However, it is important to note that our study has limitations, particularly in terms of sample size. Further research with larger cohorts is needed to explore these trends in greater depth and potentially identify specific patient subgroups that might benefit more from one treatment modality over the other. Such investigations could provide more nuanced guidance for clinical decision-making in the management of panic disorders.
